# Hydrogen sulfide toxicity inhibits primary root growth through the ROS-NO pathway

**DOI:** 10.1038/s41598-017-01046-2

**Published:** 2017-04-13

**Authors:** Ping Zhang, Qiong Luo, Ruling Wang, Jin Xu

**Affiliations:** 1 Key Laboratory of Tropical Plant Resources and Sustainable Use, Xishuangbanna Tropical Botanical Garden, Chinese Academy of Sciences, Menglun, Mengla, Yunnan 666303 China; 2 University of Chinese Academy of Sciences, Beijing, 100049 China

## Abstract

High concentrations of hydrogen sulfide (H_2_S) are toxic to plants and inhibit their growth. Previous research indicated that high concentrations of H_2_S modulate the root system architecture (RSA) by affecting auxin transport; however, the signaling pathway underlying this process remains unclear. Here, we investigated the effects of exogenous sodium hydrosulfide (NaHS), an H_2_S donor, on primary root (PR) growth in *Arabidopsis* using pharmacological, physiological, and genetic approaches. H_2_S toxicity repressed PR growth by triggering a signal transduction pathway involving reactive oxygen species (ROS) accumulation, *MITOGEN*-*ACTIVATED PROTEIN KINASE 6* (MPK6) activation, and nitric oxide (NO) production. Respiratory burst oxidase homolog mutants and an NO synthase mutant were less sensitive to NaHS, suggesting that both ROS and NO mediate the inhibitory effects of H_2_S on PR growth. We found that exogenous H_2_S-activated ROS production was required for NO generation and that *MPK6* mediated H_2_S-induced NO production. *MPK6* was shown to function downstream of ROS and upstream of NO. Finally, we demonstrated that exogenous H_2_S repressed the distribution of auxin and reduced the meristematic cell division potential in root tips, and NO was involved in this process.

## Introduction

Hydrogen sulfide (H_2_S) is a colorless gas with a characteristic odor of rotten eggs. Low concentrations of H_2_S improve the tolerance of plants to pathogens^1^, osmotic stress, salt stress, heat shock, and heavy metal stresses^2–6^. In plants, H_2_S is predominantly produced by L-cysteine desulfhydrase (DES; EC 4.4.1.1)^7^. Endogenous H_2_S plays a role in modulating plant growth and development, including seed germination, root organogenesis, stomatal closure, plant maturation and flower senescence^8–11^. Although low concentrations of H_2_S improve the tolerance of plants to abiotic and biotic stresses, high concentrations are toxic to plant growth. H_2_S toxicity-induced primary root (PR) growth inhibition has been reported^11^; however, the signaling pathway underlying H_2_S toxicity-mediated root growth and development is still unclear.

Nitric oxide (NO) is a small gas molecule that mediates lateral root (LR) formation, adventitious root growth, and root hair development^12, 13^. Our previous work indicated that NO inhibits PR growth, whereas it promotes LR development^14^. In animals, several studies have identified possible crosstalk between H_2_S and NO^15^. H_2_S physiologically modifies the cysteines in a large number of proteins via S-sulfhydration. Thus, sulfhydration appears to be a physiological posttranslational modification of proteins^16^. H_2_S increases NO production by inducing the S-sulfhydration of endothelial NO synthase (eNOS), promoting its phosphorylation, inhibiting its S-nitrosylation, and increasing eNOS dimerization (the activated form of eNOS)^17^. In plants, an interaction between H_2_S and NO in modulating plant growth and development has been reported^9, 10, 18^. H_2_S promotes NO production and acts upstream of NO to modulate abscisic acid (ABA)-dependent stomatal closure^10^. H_2_S acts upstream of indole-3-acetic acid (IAA) and NO to regulate root growth and development^9^; however, the signaling modulation mechanisms involved are largely unclear.

Mitogen-activated protein kinase (MAPK) cascades, which consist of MAPKKK (MEKK), MAPKK (MKK), and MAPK (MPK), are highly conserved signaling transduction pathways found in animals, plants and microbes^19, 20^. In plants, MAPK pathways are implicated in the regulation of growth and development and in responses to many environmental cues. The activation of MPKs alters their subcellular localization and their interactions with and phosphorylation of transcription factors, thereby reprogramming gene expression^20, 21^. *Arabidopsis* MPK3/6 are the most extensively studied MPKs in plants. Previous studies have revealed that MPK3/6 modulate plant growth, development, and stress tolerance by interacting with the ABA, ethylene, jasmonate, phosphatidic acid, Ca^2+^, and reactive oxygen species (ROS) pathways^21–26^.

Root growth and development are largely influenced by plant hormones, especially auxin^27^. Auxin is a central regulator of root formation. Auxin flux is essential for auxin to regulate of stem cell differentiation and root development^28, 29^. Auxin is an important phytohormone involved in controlling the balance between cell division and differentiation in the root meristem^30^. H_2_S-mediated root formation is alleviated by the IAA transport inhibitor N-1-naphthylphthalamic acid (NPA) and the NO scavenger 2-(4-carboxyphenyl)-4,4,5,5-tetramethylimidazoline-1-oxyl-3-oxide (cPTIO), suggesting that both IAA and NO are involved in H_2_S-mediated root system development^9^. Auxin-induced H_2_S generation is involved in LR formation in tomato^31^. Recently, Jia *et al*.^11^ showed that high levels of H_2_S inhibit auxin transport and result in alterations in root system development by modulating the polar subcellular distribution of PIN proteins^11^.

Another important pathway for regulating root system development independent of plant hormones is ROS signaling. The transcriptional regulation of ROS by the *UPBEAT1* (*UPB1*) transcription factor modulates root development by regulating cell proliferation and differentiation in root tips^30^.

In this study, using pharmacological and genetic approaches, we analyzed the possible involvement of ROS, NO, and MPK6 in exogenous H_2_S-mediated PR growth. Our results indicated that H_2_S toxicity-inhibited PR growth via the ROS-MPK6-NO signaling pathway. The potential mechanisms involved in this process are discussed.

## Results

### H_2_S toxicity inhibits PR growth by reducing the meristematic cell division potential

**Figure 1:**
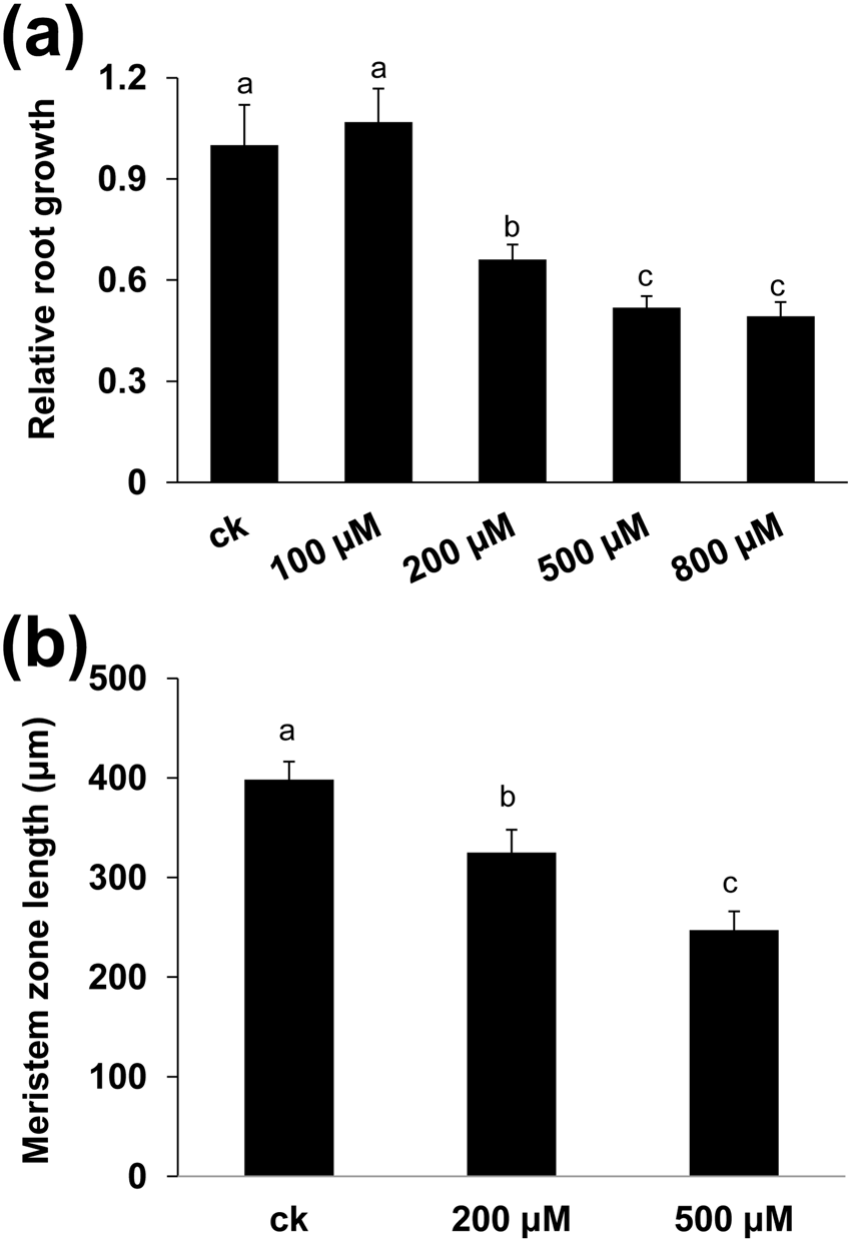
NaHS treatment inhibited PR growth. (**a**,**b**) Five-day-old wild-type seedlings grown in 1/2 MS medium were treated with 100–800 μM NaHS for 2 d, and (**a**) PR growth and (**b**) the length of the meristem zone were measured after treatment. ck, untreated control. n = 60. Error bars represent ± SD. Different letters indicate significantly different values (P < 0.05 by Tukey’s test).

Five-day-old seedlings germinated on 1/2 MS medium were transferred to fresh medium with 0–800 μM NaHS, an H_2_S donor, and PR growth was measured 2 d after transfer to determine how exogenous H_2_S affects PR growth. With increasing NaHS concentrations of 200 μM to 800 μM, PR growth was inhibited by 34% to 51% (Fig. 1a, Fig. S1). To examine the inhibitory effects of H_2_S on PR growth, we also measured the length of the meristem zone in H_2_S-treated roots. As shown in Fig. 1b, the lengths of the meristematic zones decreased by 27% and 36.6% in roots treated with 200 μM and 800 μM exogenous NaHS, respectively. Because treatment with 500 μM NaHS induced an approximately 50% decrease in PR growth, we selected this concentration for use in subsequent experiments.

### ROS are involved in H_2_S toxicity-induced PR growth inhibition

**Figure 2:**
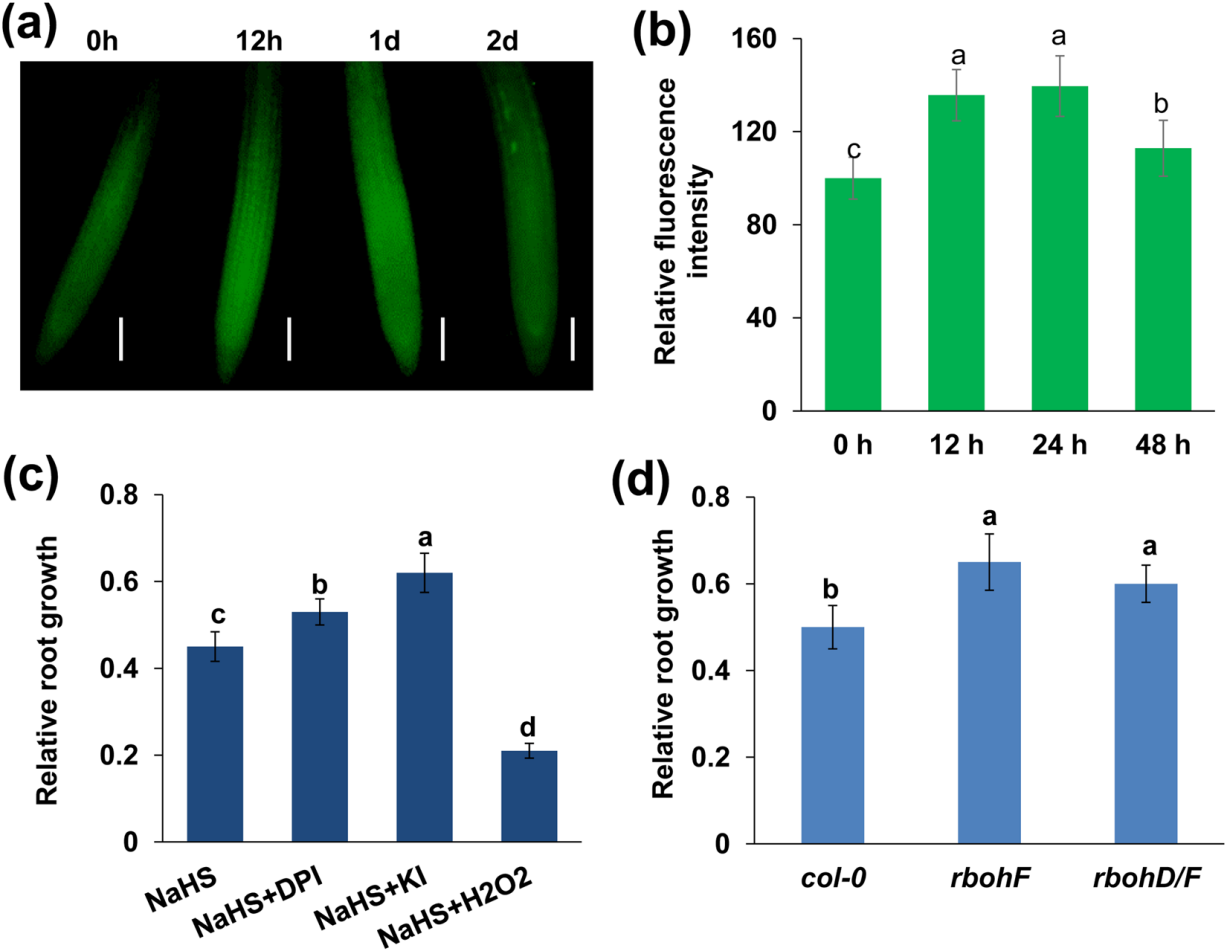
NaHS induces the accumulation of ROS. (**a**,**b**) Detection of ROS production in the roots of 5-d-old *Col*-*0* seedlings exposed to 500 μM NaHS for periods of up to 2 d using the ROS-specific fluorescent probe DCFH-DA (**a**) and quantification of ROS-specific fluorescence intensities (**b**) in plants treated as described in (**a**). The fluorescence intensity of the untreated roots was set to 100. Bars, 100 μm. n = 30. (**c**) Relative root growth of *Col*-*0* seedlings treated with 500 μM NaHS in the presence or absence of 1 μM DPI, 1 mM KI, and 1 mM H_2_O_2_ for 2 d compared with untreated seedlings. (**d**) Relative root growth of *col*-*0*, *rbohF*, and *rbohD*/*F* seedlings treated with 500 μM NaHS for 2 d compared with untreated seedlings. n = 45. Error bars represent ± SD. Different letters indicate significantly different values (P < 0.05 by Tukey’s test).

Two independent signaling pathways regulate root growth and development: auxin signaling and the ROS signaling pathway^30^. Jia *et al*.^11^ found that H_2_S toxicity inhibits auxin transport and results in alterations in root system development^11^. To investigate whether ROS are also involved in H_2_S toxicity-induced PR growth inhibition, we assessed ROS production in the roots using the ROS-specific-fluorescent probe 2,7-dichlorofluorescin diacetate (DCFH-DA). Exogenous H_2_S increased ROS levels (Fig. 2a and b) in the roots; this increase was sustained for 24 h, after which ROS production gradually declined.

To further assess whether ROS participate in the H_2_S-mediated inhibition of root growth, we next observed the effects of diphenylene iodonium (DPI), an inhibitor of NADPH oxidase, and potassium iodide (KI), an ROS scavenger, on the H_2_S-mediated inhibition of root growth in the roots of wild-type plants. DPI and KI alleviated, whereas exogenous H_2_O_2_ increased he H_2_S-induced inhibition of root growth (Fig. 2c, Fig. S2). To corroborate these pharmacological data, we further used the *Arabidopsis* ROS-biosynthesis-related respiratory burst oxidase homologue (Rboh) NADPH oxidase single and double mutants *rbohF* and *rbohD*/*F* and found that PR growth was 31% and 20% greater in *rbohF* and *rbohD*/*F* than in wild-type plants subjected to exogenous H_2_S treatment (Fig. 2d and e). These data confirm the essential role of ROS in the modulation of root growth by H_2_S.

### NO is also required for the inhibition of PR growth by exogenous H_2_S

**Figure 3:**
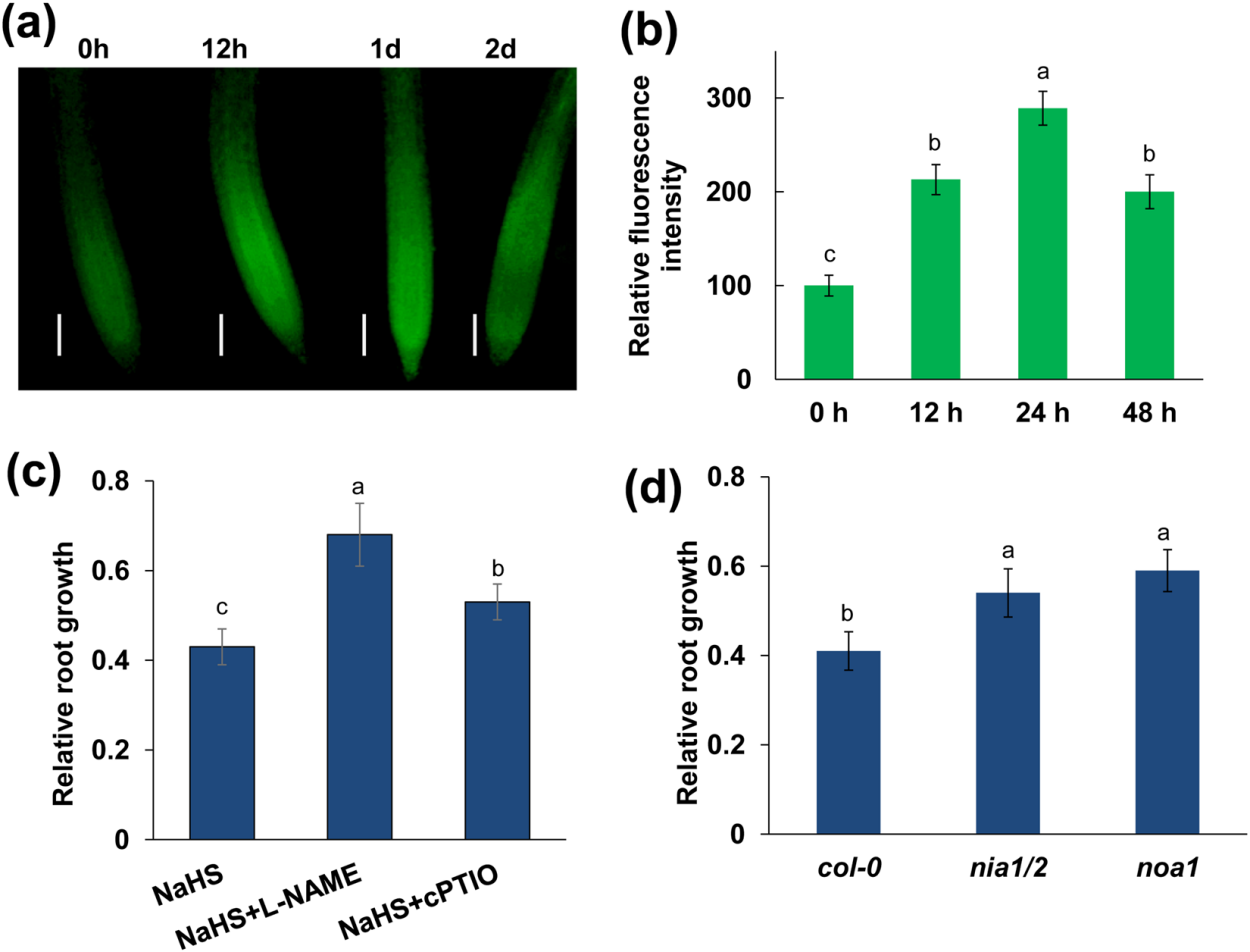
NO is involved in the NaHS-mediated inhibition of PR growth. (**a**,**b**) Detection of NO production in the roots of 5-d-old wild-type seedlings exposed to 500 μM NaHS for periods of up to 2 d using the NO-specific fluorescence probe DAF-2 DA (**a**) and quantification of NO-specific fluorescence intensities (**b**) in plants treated as described in (**a**). The fluorescence intensity of the untreated roots was set to 100. Bars, 100 μm. n = 30. (**c**) Relative root growth of *Col*-*0* seedlings treated with 500 μM NaHS in the presence or absence of 500 μM L-NAME and 200 μM cPTIO for 2 d compared with untreated seedlings. (**d**) Relative root growth of *Col*-*0*, *nia1*/*2*, and *noa1* seedlings treated with 500 μM NaHS for 2 d compared with untreated seedlings. n = 45. Error bars represent ± SD. Different letters indicate significantly different values (P < 0.05 by Tukey’s test).

NO is another important gaseous signaling molecule that mediates root development^12, 13^. We therefore investigated NO production in the roots using the NO-specific fluorescent probe, 4,5-diaminofluorescein diacetate (DAF-2DA). Similar to its effects on ROS production, exogenous H_2_S triggered an increase in NO production in the roots; this increase was sustained for 24 h, after which NO production gradually declined (Fig. 3a and b). We further examined whether NO is involved in H_2_S-mediated PR growth inhibition. As shown in Fig. 3c and Fig. S3, supplementation with the NO synthase (NOS) inhibitor *N* ^*G*^-nitro-L-Arg-methyl ester (L-NAME), or the NO scavenger 2-(4-carboxyphenyl)-4,4,5,5-tetramethylimidazoline-1-oxyl-3-oxide (cPTIO) alleviated the NaHS-induced inhibition of root growth by 54.7% and 21.9%, respectively. Consistent with these pharmacological results, the NO biosynthesis-related *nia1*-*2*/*2*-*5* double mutant and the *noa1* mutant were less sensitive to exogenous H_2_S treatment than were wild-type plants (Fig. 3d and e).

**Figure 4:**
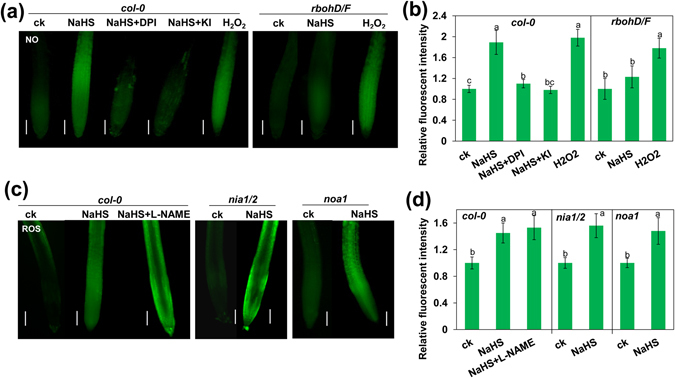
(**a**,**b**) Detection of NO production in the roots of 5-d-old wild-type *Col*-*0* and *rbohD*/*F* seedlings exposed to 500 μM NaHS with or without 1 μM DPI, 1 mM KI, and 1 mM H_2_O_2_ for 24 h using the NO-specific fluorescent probe DAF-2 DA (**a**) and quantification of NO-specific fluorescence intensities (**b**) in plants treated as described in (**a**). (**c**,**d**) Detection of H_2_O_2_ production in the roots of 5-d-old wild-type *Col*-*0*, *nia1*/*2*, and *noa1* seedlings exposed to 500 μM NaHS with or without 500 μM L-NAME for 24 h using the ROS-specific fluorescent probe DCFH-DA (**c**) and quantification of H_2_O_2_-specific fluorescence intensities (**d**) in plants treated as described in (**c**). The fluorescence intensity of the untreated roots was set to 100. Bars, 100 μm. n = 30. ck, untreated control. Error bars represent ± SD.

We found that supplementation with KI or DPI markedly inhibited NO production compared with that of the exogenous H_2_S treatment alone in the roots (Fig. 4a and b), whereas supplementation with L-NAME did not alter ROS levels in exogenous H_2_S-treated roots (Fig. 4c and d). To further confirm the relationship between ROS and NO in the exogenous H_2_S-mediated inhibition of root growth, we assessed the *rbohD*/*F*, *nia1*-*2*/*2*-*5*, and *noa1* mutants. We found that exogenous H_2_S-induced NO production was abolished in the *rbohD*/*F* double mutant, while supplementation with H_2_O_2_ markedly induced NO production in both the *Col*-*0* and the *rbohD*/*F* double mutant (Fig. 4a and b). Meanwhile, H_2_S-induced ROS production was similar in the *col*-*0*, *nia1*-*2*/*2*-*5*, and *noa1* lines (Fig. 4c and d). These data indicate that ROS is involved in exogenous H_2_S-induced NO production in the roots.

**Figure 5:**
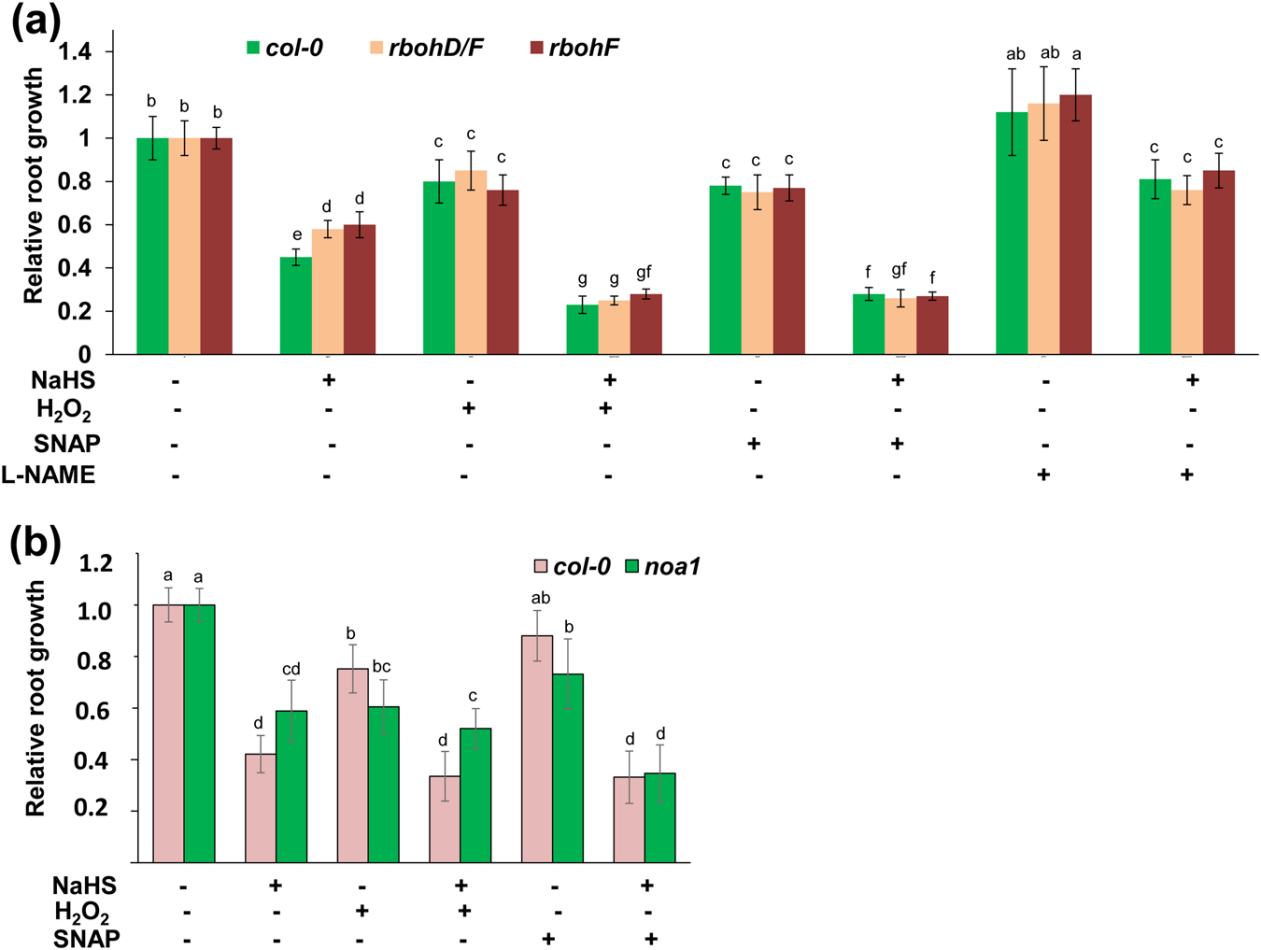
(**a**) Relative root growth of *Col*-*0*, *rbohF*, and *rbohD*/*F* seedlings exposed to 500 μM NaHS with or without 1 mM H_2_O_2_, 100 μM SNAP, and 500 μM L-NAME for 2 d compared with untreated seedlings. (**b**) Relative root growth of *Col*-*0* and *noa1* seedlings exposed to 500 μM NaHS with or without 1 mM H_2_O_2_ and 100 μM SNAP for 2 d compared with untreated seedlings. n = 45. Error bars represent ± SD. Different letters indicate significantly different values (P < 0.05 by Tukey’s test).

We also found that supplementation with the NO donor SNAP completely reversed the insensitivity of the *rbohF*, *rbohD*/*F*, and *noa1* mutants to exogenous H_2_S (Fig. 5a and b). Additionally, supplementation with H_2_O_2_ completely reversed the insensitivity of the *rbohF* and *rbohD*/*F* mutants to exogenous H_2_S (Fig. 5a), but did not rescue the insensitivity of the *noa1* mutant (Fig. 5b). Taken together, these data suggest that exogenous H_2_S-induced ROS production functions upstream of NO accumulation in the roots.

### MPK6 mediates the inhibition of PR growth by exogenous H_2_S

**Figure 6:**
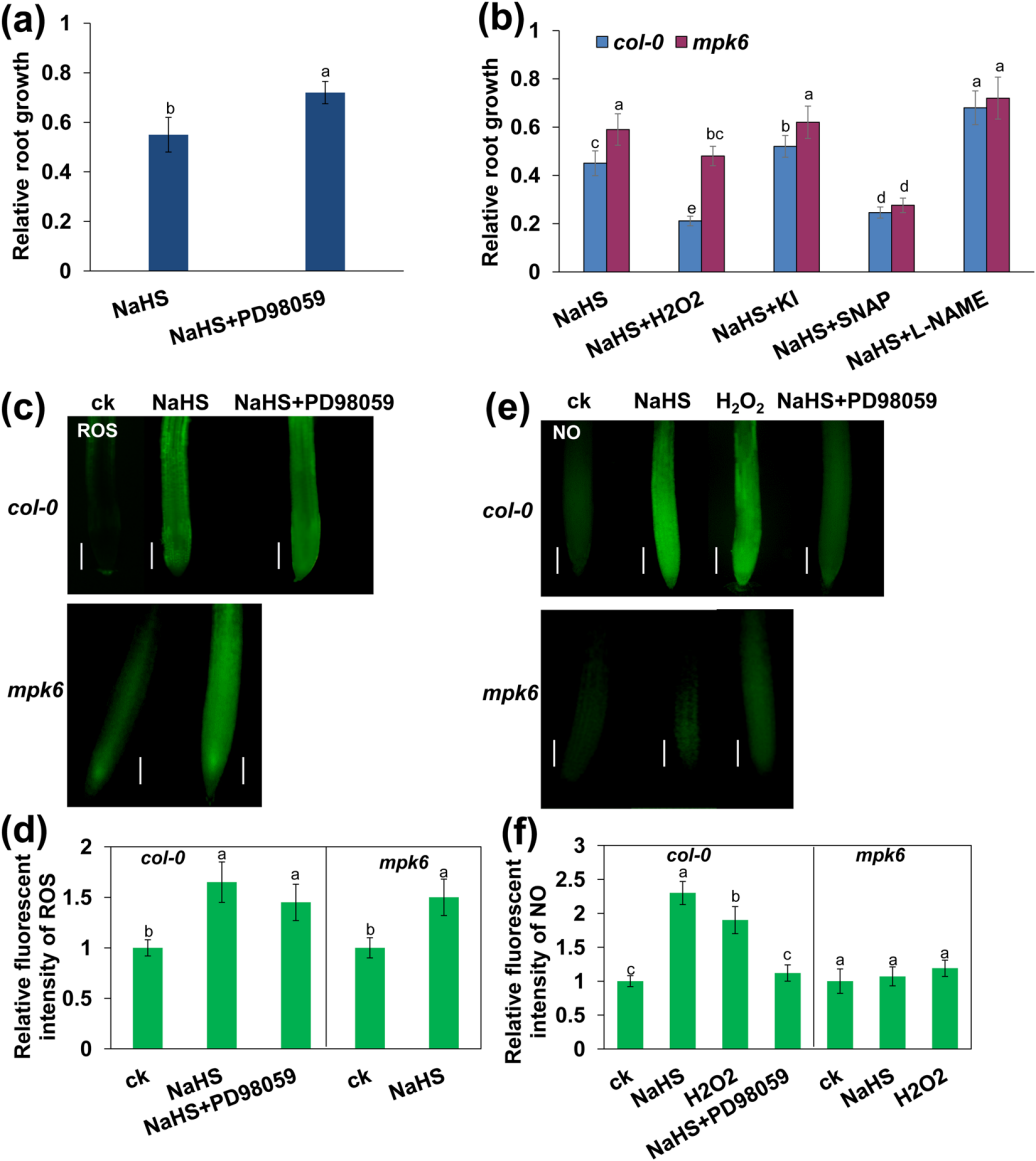
MPK6 mediates PR growth inhibition by NaHS. (**a**) Relative root growth of *Col*-*0* seedlings treated with 500 μM NaHS in the presence or absence of 150 μM PD98059 for 2 d compared with untreated seedlings. (**b**) Relative root growth of *Col*-*0* and *mpk6* seedlings exposed to 500 μM NaHS with or without 1 mM H_2_O_2_, 100 μM SNAP, 1 mM KI, and 500 μM L-NAME for 2 d compared with untreated seedlings. n = 45. (**c**,**d**) Detection of ROS production in the roots of 5-d-old wild-type *col*-*0* and *mpk6* seedlings exposed to 500 μM NaHS with or without 150 μM PD98059 for 24 h by the ROS-specific fluorescent probe DCFH-DA (**c**) and the quantification of the ROS-specific fluorescence intensities (**d**) in plants treated as described in (**c**). (**e**,**f**) Detection of NO production in the roots of 5-d-old wild-type *col*-*0* and *mpk6* seedlings exposed to 500 μM NaHS with or without 1 mM H_2_O_2_ or 150 μM PD98059 for 24 h using the NO-specific fluorescence probe DAF-2 DA (**e**) and quantification of NO-specific fluorescence intensities (**f**) in plants treated as described in (**e**). n = 30. Bars, 100 μm. ck, untreated control. Error bars represent ± SD. Different letters indicate significantly different values (P < 0.05 by Tukey’s test).

Previous studies have indicated that MAPK is involved in the ROS signaling pathway^21, 24, 25^. To determine whether MAPK proteins mediate exogenous H_2_S-induced PR growth inhibition, we first observed the effects of PD98059, an inhibitor of MAPK, on PR growth in wild-type *Col*-*0* plants. As shown in Fig. 6a and Fig. S4, supplementation with PD98059 alleviated the exogenous H_2_S-induced inhibition of PR growth by 29.6%, suggesting that MAPK is involved in the modulation of PR growth by exogenous H_2_S in *Arabidopsis*. To confirm the role of MAPK in the exogenous H_2_S-mediated inhibition of root growth, we used the *Arabidopsis* mutant *mpk6*. As shown in Fig. 6b, the inhibition of PR growth by exogenous H_2_S was alleviated by 37% in the *mpk6* mutant compared with *Col*-*0* seedlings. These data provide genetic evidence showing that MPK6 plays an important role in the inhibition of PR growth by exogenous H_2_S. Together with the pharmacological data described above, these results clearly indicate that MPK6 is a positive regulator that mediates the exogenous H_2_S-induced inhibition of PR growth in *Arabidopsis*.

### MPK6 promotes NO production downstream of ROS in the exogenous H_2_S-mediated inhibition of PR growth

The above results suggested that ROS, NO, and MPK6 are important intermediate signaling molecules in the modulation of PR growth by H_2_S. We then examined the relationship between ROS, NO, and MPK6 in the exogenous H_2_S-mediated inhibition of PR growth. For this purpose, we measured the production of ROS and NO in roots treated with exogenous H_2_S. In H_2_S-treated roots, the MAPK inhibitor PD98059 did not affect ROS production, but it markedly inhibited NO production (Fig. 6c–f). Additionally, in the roots of the *mpk6* mutant compared with those of *Col*-*0*, H_2_S treatment did not affect ROS production, but NO production was abolished (Fig. 6c–f). ROS-induced NO production was also repressed in the *mpk6* mutant (Fig. 6e and f). Supplementation with KI alleviated the H_2_S-mediated inhibition of PR growth by 16% in *Col*-*0* roots but could not further alleviate the H_2_S-mediated inhibition of PR growth in *mpk6* roots. In contrast, supplementation with H_2_O_2_ increased the H_2_S-mediated inhibition of PR growth by 51% in the *Col*-*0* roots; however, the inhibitory effects of H_2_O_2_ on PR growth were weaker in *mpk6* seedlings than in *Col*-*0* seedlings exposed to exogenous H_2_S (Fig. 6b). These weaker effects may have occurred because the loss of MPK6 in the *mpk6* mutant resulted in the inhibition of the ROS signaling transduction pathway.

We next analyzed the effects of NO on PR growth inhibition in the *mpk6* mutant and found that supplementation with the NO donor SNAP increased the inhibitory effects of H_2_S on PR growth, while supplementation with L-NAME reduced these effects in both *mpk6* and *Col*-*0* seedlings (Fig. 6b). Compared with *Col*-*0* plants, supplementation with SNAP or L-NAME rescued the insensitivity of the *mpk6* mutant to exogenous H_2_S-induced PR growth inhibition (Fig. 6b). These data indicated that MPK6 mediated inhibition of PR growth downstream of ROS and upstream of NO.

### NO is involved in exogenous H_2_S-mediated inhibition of PR growth by regulating auxin distribution in root tips

**Figure 7:**
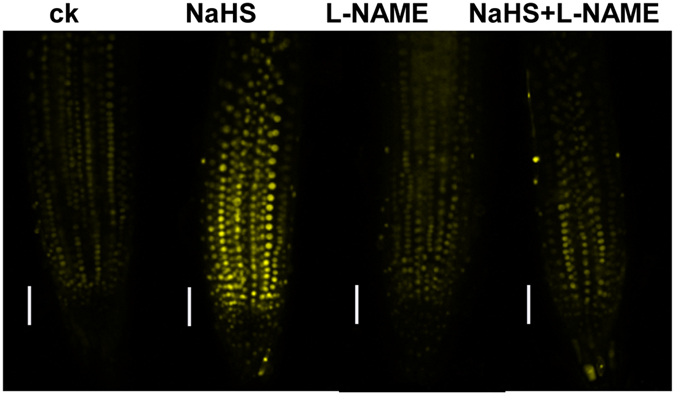
NO is involved in the NaHS-mediated reduction of the distribution of auxin in root tips. YFP fluorescence in the roots of 5-d-old *DII*-*VENUS* seedlings exposed to 500 μM NaHS with or without 500 μM L-NAME for 24 h. Bars, 50 μm. n = 30. ck, untreated control.

Auxin plays a central role in modulating root growth and development^32^. Previous work indicated that H_2_S toxicity reduced auxin accumulation in root tips^11^. We therefore investigated whether H_2_S-induced NO production is involved in the regulation of auxin distribution. For this purpose, we used an auxin-perceptive *DII*-*VENUS* line to monitor the possible changes in auxin distribution in H_2_S-treated plants in the presence or absence of the NOS inhibitor L-NAME. Consistent with the result of Jia *et al*.^11^, our data here showed that the DII-VENUS fluorescence were higher in NaHS-treated roots than in untreated plants^11^. Supplementation with L-NAME decreased the DII-VENUS fluorescence in H_2_S-treated seedlings (Fig. 7a), suggesting that H_2_S-induced NO production affects the distribution of auxin.

## Discussion

At low concentrations, H_2_S is an important regulator of the stress response, which is essential for stress tolerance and survival in plants^3, 5, 6^. However, high concentrations of H_2_S are toxic to plants and inhibit their growth. A recent investigation indicated that high concentrations of H_2_S inhibit root growth by regulating auxin accumulation in root tips^11^; however, the molecular mechanisms underlying this process are largely unclear. In the present study, we elucidated a signaling pathway that controls H_2_S toxicity-induced PR growth inhibition in *Arabidopsis*. We showed that the activation of MPK6, NADPH oxidase-dependent H_2_O_2_ synthesis, Nia1/NOA1-dependent NO production, and the regulation of auxin perception are all required for the inhibition of PR growth by exogenous H_2_S. Furthermore, we found that exogenous H_2_S-induced NO production is mediated by ROS and that the modulation of NO production by ROS required the activation of MPK6. We also showed that exogenous H_2_S-mediated changes in auxin distribution were regulated by NO. Therefore, our study elucidates an H_2_S toxicity-mediated root growth signaling pathway that involves a cascade of NADPH oxidase-dependent ROS production, which, in turn, leads to *MPK6*- and *Nia1*/*NOA1*-dependent NO production.

### H_2_S toxicity-stimulated ROS production inhibits PR growth

Previous reports have shown that ROS are involved in the modulation of PR growth and LR formation by phytohormones or environmental cues^10, 14, 18, 33^. There are different ROS sources in plants, including cell wall peroxidases, NADPH oxidases, and amine oxidase-type enzymes^34–36^. In this study, we found that exogenous H_2_S-induced ROS production was markedly inhibited by the NADPH oxidase inhibitor DPI and that the NADPH oxidase null mutants *rbohF* and *rbohD*/*F* were defective in exogenous H_2_S-induced ROS generation and showed a smaller reduction in PR growth than did wild-type plants. In contrast, supplementation with H_2_O_2_ further increased H_2_S-induced inhibition of root growth. These results indicated that exogenous H_2_S induces ROS generation in the roots and inhibits subsequent PR growth.

### ROS-activated MPK6 promotes NO production in H_2_S-treated plants

In plants, MPKs are important signaling molecules that are activated in response to a variety of environmental and developmental cues^21, 26^. In this study, we showed that MPK6 plays an important role in the regulation of the H_2_S toxicity-mediated inhibition of root growth. The MAPK inhibitor PD98059 alleviated the H_2_S-induced inhibition of PR growth, indicating that the activation of MAPK is required for this inhibition. The *mpk6* mutant showed reduced inhibition of PR growth following exogenous H_2_S treatment, providing genetic evidence for the essential role of MPK6 in H_2_S toxicity-mediated root growth.

Previous studies have indicated that *MPK6* modulates plant growth and the response to stimuli by interacting with ROS and/or NO^26^. NO promots Cd^2+^-induced programmed cell death (PCD) by enhancing MPK6-mediated caspase-3-like activation in *Arabidopsis* ^37^. *MPK6* controls H_2_O_2_-induced root growth by mediating the Ca^2+^ influx in *Arabidopsis* ^26^. The *mpk6* mutant produces more and LRs than do wild-type plants after application of the NO donor sodium nitroprusside (SNP) or H_2_O_2_^38^. These studies indicate that *MPK6* modulates NO production and the response to ROS during root development; however, it is unknown whether and how the interactions between MPK6, ROS, and NO mediate the H_2_S toxicity-induced inhibition of PR growth. In this study, we found that the H_2_S toxicity-induced production of ROS and NO and inhibition of PR growth were markedly impaired in the *rbohF* and *rbohD*/*F* mutants. Moreover, the H_2_S-induced increase in NO and inhibition of PR growth were also impaired in the *mpk6*, *nia1*-*2*/*2*-*5*, and *noa1* mutants. ROS rescued the defect in the H_2_S-induced inhibition of PR growth in the *rbohF*, *rbohD*/*F*, *mpk6*, *nia1*-*2*/*2*-*5*, and *noa1* mutants, while only NO could rescue the defect in the H_2_S-induced inhibition of PR growth in the *nia1*-*2*/*2*-*5* and *noa1* mutants. These data confirmed that ROS-activated MPK6 promoted NO production and thereby reduced PR growth in H_2_S-treated plants. Our results show that ROS-activated MPK6 and NO production are involved in the H_2_S toxicity-mediated inhibition of PR growth and suggest that the ROS-MPK6-NO pathway is a general signaling transduction pathway that regulates plant responses to abiotic stress. The phosphorylation of NIA2 by MPK6 leads to increases in nitrate reductase (NR) activity and NO production^38^. However, how MPK6 mediates NO production via the NO synthase-associated (NOA) pathway remains unclear.

### H_2_S toxicity-induced NO production inhibits PR growth

Similar to its effect on ROS, we found that H_2_S toxicity triggered NO accumulation in root tips within 24 h, which gradually decreased after 48 h of treatment, suggesting that both ROS and NO could act as early signaling molecules to modulate downstream gene expression, ultimately leading to PR growth inhibition.

It has been proposed that there are multiple sources of NO generation in plant cells, including NR^39^, the NO synthase-like/NO synthase-associated (NOS-like/NOA) enzyme^40^, and non-enzymatic pathways^41^. The roles of the NOA pathway and the NR pathway in NO generation *in vivo* have been well studied. Previous reports have shown that L-NAME, an L-arginine analog, inhibits NOS activity in plants^42^. In this study, we found that supplementation with the NOS inhibitor L-NAME markedly repressed the production of NO in H_2_S-treated roots. Our genetic experiments also showed that H_2_S-induced NO production and PR growth inhibition were markedly impaired in the *NOA1*-defective mutant *noa1* and the *NR*-defective *nia1*-*2*/*2*-*5* double mutant. These results suggested that NO production in H_2_S-treated seedlings may be catalyzed through both L-Arg-dependent and NR-dependent routes. Interestingly, Lisjak *et al*.^43^ found that the exogenous H_2_S donor NaHS repressed the ABA-induced accumulation of NO in guard cells^43^. The differences in these effects between studies may be due to the different tissues examined.

Previous studies have indicated that NO acts as a second messenger to regulate root growth via the auxin pathway^44, 45^. This prompted us to assess the possible involvement of NO in the H_2_S-mediated auxin distribution. Jia *et al*.^11^ reported that H_2_S reduces auxin accumulation in root tips by disrupting the expression of auxin carriers and subsequent polar auxin transport (PAT)^11^. Consistent with these findings, our data indicated that H_2_S toxicity increased DII-VENUS fluorescence in roots, while supplementation with L-NAME decreased DII-VENUS fluorescence. These data suggest that NO accumulation is responsible for the H_2_S-induced inhibition of the distribution of auxin in root tips.

**Figure 8:**
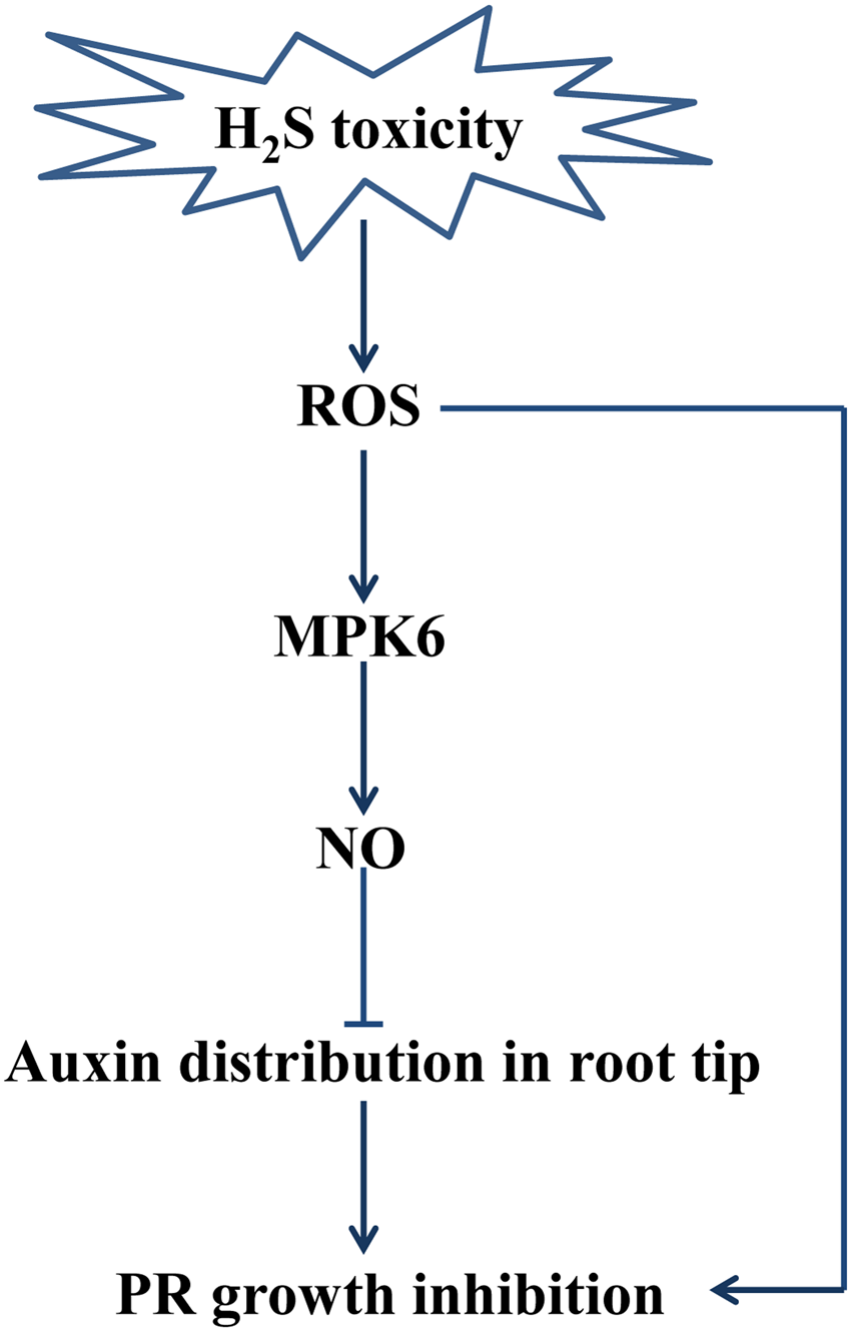
H_2_S toxicity inhibits PR growth via the ROS-MPK6-NO signaling pathway. High-concentration H_2_S induced ROS production via the NADPH oxidase pathway, which directly inhibited PR growth and activated MPK6. MPK6 then promoted NO production through both L-Arg-dependent and NR-dependent routes. Elevated NO repressed auxin distribution, ultimately inhibiting PR growth.

Taken together, our data indicate that in addition to the auxin pathway, ROS and NO are also key players in the plant response to H_2_S toxicity. ROS production in roots induced by a high concentration of H_2_S inhibited PR growth, while ROS accumulation in roots activated MPK6. MPK6 then promoted NO production through both L-Arg- and NR-dependent routes. Elevated NO repressed the distribution of auxin in root tips, thereby reducing PR growth (Fig. 8). These results indicate that H_2_S toxicity inhibits PR growth via both the ROS pathway and the auxin signaling pathway. These findings show that the ROS-MPK6-NO signaling pathway mediates plant responses to H_2_S toxicity through morphological and physiological changes in the roots.

## Methods

### Plant growth and chemical treatments

The *Arabidopsis thaliana* ecotype *Columbia*-*0* was used in this study. The transgenic and mutant lines employed in this work included *DII*-*YFP*, *nia1*/*2*, *noa1*, *rbohF*, *rbohD*/*F*, and *mpk6*. Seeds were surface sterilized with 5% bleach for 5 min, then washed five times with sterile water, incubated for 2 d at 4 °C in the dark and plated onto agar medium containing half-strength MS (Sigma), pH5.70, supplemented with 1% agar and 10% sucrose. Seedlings were grown in a growth chamber maintained at 22 °C under a 16/8 h light/dark cycle. Seeds were grown in the vertical position. Five-day-old seedlings were transferred to plates supplemented with various chemicals, including sodium hydrosulfide (NaHS), cPTIO, L-NAME, DPI, KI, SNAP, H_2_O_2_, and PD98059, and grown for an additional 2 d. All chemicals were obtained from Sigma-Aldrich.

### Phenotypic analysis

Seeds were grown in the vertical position. After the seedlings were transferred to plates supplemented with various chemicals, root growth was measured at the same time every day. After 5 d of treatment, the PR length was measured and statistically analyzed. The length of the meristematic zone was measured from the quiescent center (QC) to the beginning of the root elongation zone as described by Liu *et al*.^32^. At least 15 replicate plants were measured for each treatment.

### Measurement of NO and ROS production and fluorescence microscopy

The endogenous NO levels in root meristems were visualized using the specific NO fluorescent probe DAF-2 DA. Seedlings were incubated at 37 °C in a 5 μM staining solution for 1 h. The endogenous ROS levels in root meristems were visualized using the specific ROS fluorescent probe DCFH- DA. Seedlings were incubated at 37 °C in 10 μM staining solution for 5 min. Then, they were washed twice and viewed under a microscope. Quantitative measurement of fluorescence intensity was performed using ImageJ.

Roots were treated with DAF-2 DA and DCFH-DA and analyzed using fluorescence microscopy (Zeiss; NO: excitation 495 nm, emission 515 nm; ROS: excitation 488 nm, emission 525 nm). DII-VENUS fluorescence was observed using confocal laser scanning microscopy (Zeiss) according to the manufacturer’s instructions. The excitation and emission wavelengths were 488 to 520 nm for YFP.

### Statistical analysis

For the PR growth and fluorescence microscopy analysis, the experiments were repeated three times with 15–20 seedlings in each repeat. The data were analyzed using Image-Pro Plus software (version 4.5.1.29; Media Cybernetics, Carlsbad, CA) and SPSS (Statistical Package for the Social Sciences) software. The results are presented as the mean ± SD. For statistical analysis, we used Tukey’s test (*P* < 0.05)^46, 47^.

## Electronic supplementary material

Additional information
