# Supplementary material for: Hydrogen sulfide toxicity inhibits primary root growth through the ROS-NO pathway
Source: Sci Rep. 2017 Apr 13;7:868. doi: 10.1038/s41598-017-01046-2 (PMC5429837; doi:10.1038/s41598-017-01046-2)
Supplement: Supplementary file 1 — Additional information [file 41598_2017_1046_MOESM1_ESM.pdf]

Additional information

Hydrogen sulfide toxicity inhibits primary root growth through the ROS-NO pathway

Ping Zhang[1,2†], Qiong Luo[1†], Ruling Wang[1] & Jin Xu[1*]

[1] Key Laboratory of Tropical Plant Resources and Sustainable Use, Xishuangbanna Tropical Botanical Garden, Chinese Academy of Sciences, Menglun, Mengla, Yunnan 666303, China

[2] University of Chinese Academy of Sciences, Beijing, 100049, China

[†] These authors have contributed equally to this work.

Correspondence and requests for materials should be addressed to J.X.

(xujin@xtbg.ac.cn)

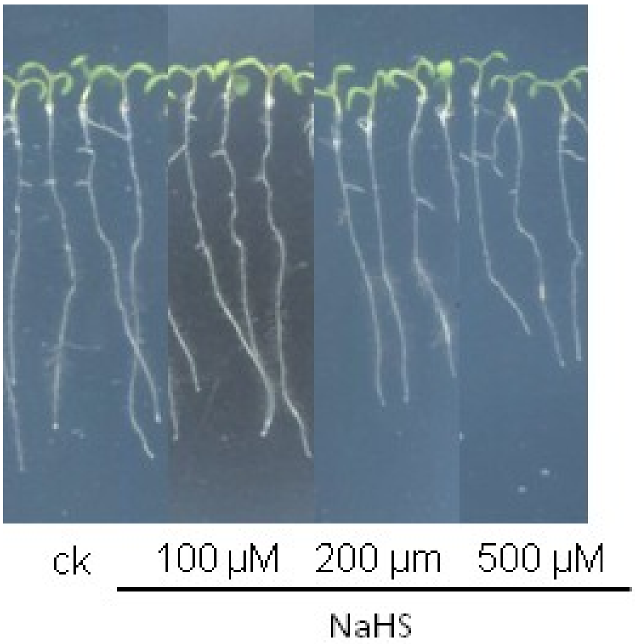

Figure S1 NaHS treatment inhibited PR growth. Five-day-old wild-type seedlings grown in 1/2 MS medium were treated with 100, 200, and 500 μM NaHS for 24 h, and the PR growth were measured after treatment.

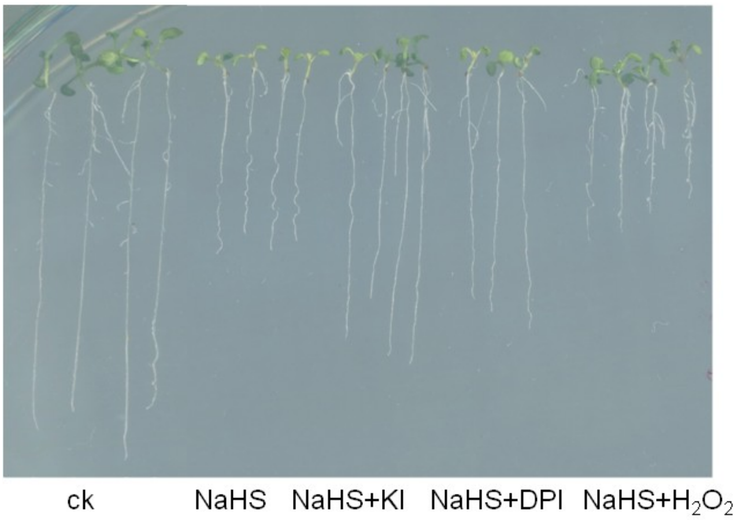

Figure S2 PR growth of *col-0* seedlings treated with 500 μM NaHS in the presence or absence of 1 mM KI, 1 μM DPI, and 1 mM H_2O_2 for 2 d.

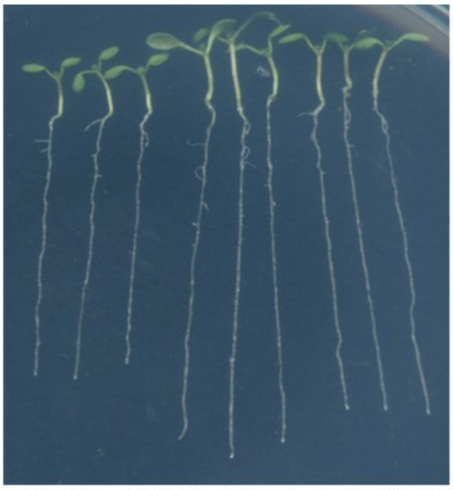

Figure S3 PR growth of *col-0* seedlings exposed to 500 μM NaHS with or without 1 mM H_2O_2, 100 μM SNAP, 1 mM KI, and 500 μM L-NAME for 2 d.

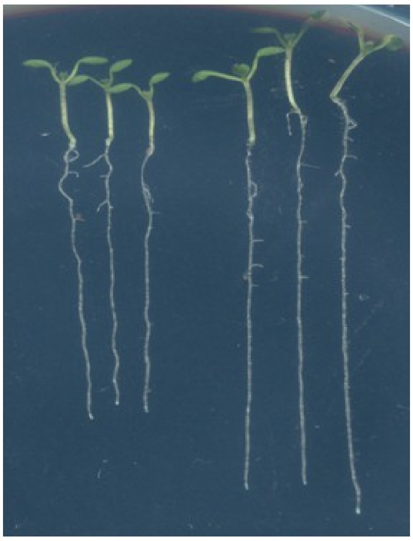

Figure S4 PR growth of *col-0* seedlings treated with 500 μM NaHS in the presence or absence of 150 μM PD98059 for 2 d
